# Ultra-high definition (8K UHD) endoscope: our first clinical success

**DOI:** 10.1186/s40064-016-3135-z

**Published:** 2016-08-30

**Authors:** Hiromasa Yamashita, Hisae Aoki, Kenkichi Tanioka, Toshiyuki Mori, Toshio Chiba

**Affiliations:** 1 University Research Center, Nohon University, 1-7-3, Kanda Surugadai, Chiyoda-ku, Tokyo, 101-0062 Japan; 2 Medical Imaging Consortium, 5-5, Shinogawa-machi, Shinjuku-ku, Tokyo, 162-0814 Japan; 3 Faculty of Medicine, Kyorin University, 6-20-2, Shinkawa, Mitaka-shi, Tokyo, 181-0004 Japan

**Keywords:** 8K ultra-high definition (UHD), 4K ultra-high definition (UHD), Laparoscopic surgery, Cholecystectomy

## Abstract

**Background:**

We have started clinical application of 8K ultra-high definition (UHD; 7680 × 4320 pixels) imaging technology, which is a 16-fold higher resolution than the current 2K high-definition (HD; 1920 × 1080 pixels) technology, to an endoscope for advanced laparoscopic surgery.

**Results:**

Based on preliminary testing experience and with subsequent technical and system improvements, we then proceeded to perform two cases of cholecystectomy and were able to achieve clinical success with an 8K UHD endoscopic system, which consisted of an 8K camera, a 30-degrees angled rigid endoscope with a lens adapter, a pair of 300-W xenon light sources, an 85-inch 8K LCD and an 8K video recorder. These experimental and clinical studies revealed the engineering and clinical feasibility of the 8K UHD endoscope, enabling us to have a positive outlook on its prospective use in clinical practice.

**Conclusions:**

The 8K UHD endoscopy promises to open up new possibilities for intricate procedures including anastomoses of thin nerves and blood vessels as well as more confident surgical resections of a diversity of cancer tissues. 8K endoscopic imaging, compared to imaging by the current 2K imaging technology, is very likely to lead to major changes in the future of medical practice.

## Background

The present burgeoning of 8K ultra-high definition (UHD) imaging technology, ushering in an era of video with a 16-fold higher resolution (7680 × 4320 pixels, about 33 million pixels) than the current high-definition (HD, 1920 × 1080 pixels, about 2 million pixels) technology, is enabling the development of the next generation 8K broadcasting system (Sugawara et al. 2003, 2013; Yamashita et al. 2012; Shimamoto et al. 2014). In this context, there are heightening expectations in recent years for much important contributions of 8K UHD technology to innovative medical imaging in advanced image-guided diagnosis and treatment. Between HD and 8K UHD 4K UHD imaging technology (3840 × 2160 pixels, about 8 million pixels) is gradually progressed for medical use (International Telecommunication Union (ITU) 2012; Woo 2016; Maier et al. 2015), however, we started by developing an 8K UHD camera specifically designed for endoscopic use, and subsequently confirmed its capability of 8K imaging resolution in use coupled with a rigid endoscope. Based on preliminary testing experience and with subsequent technical and system improvements, we then proceeded to perform two cases of cholecystectomy and were able to achieve clinical success in the full employment of an 8K UHD endoscopic system for the first time in the world. These experimental and clinical studies revealed the mechanical and technical feasibility of the 8K UHD endoscope, enabling us to have a positive outlook on its prospective use in clinical practice. In this paper, we report on the outcome of our feasibility study of our current 8K UHD endoscopy, including its system configuration as well as the problems that need to be overcome before expanding its use in clinical practice.

## Methods

### System configuration of the 8K UHD endoscope

**Fig. 1:**
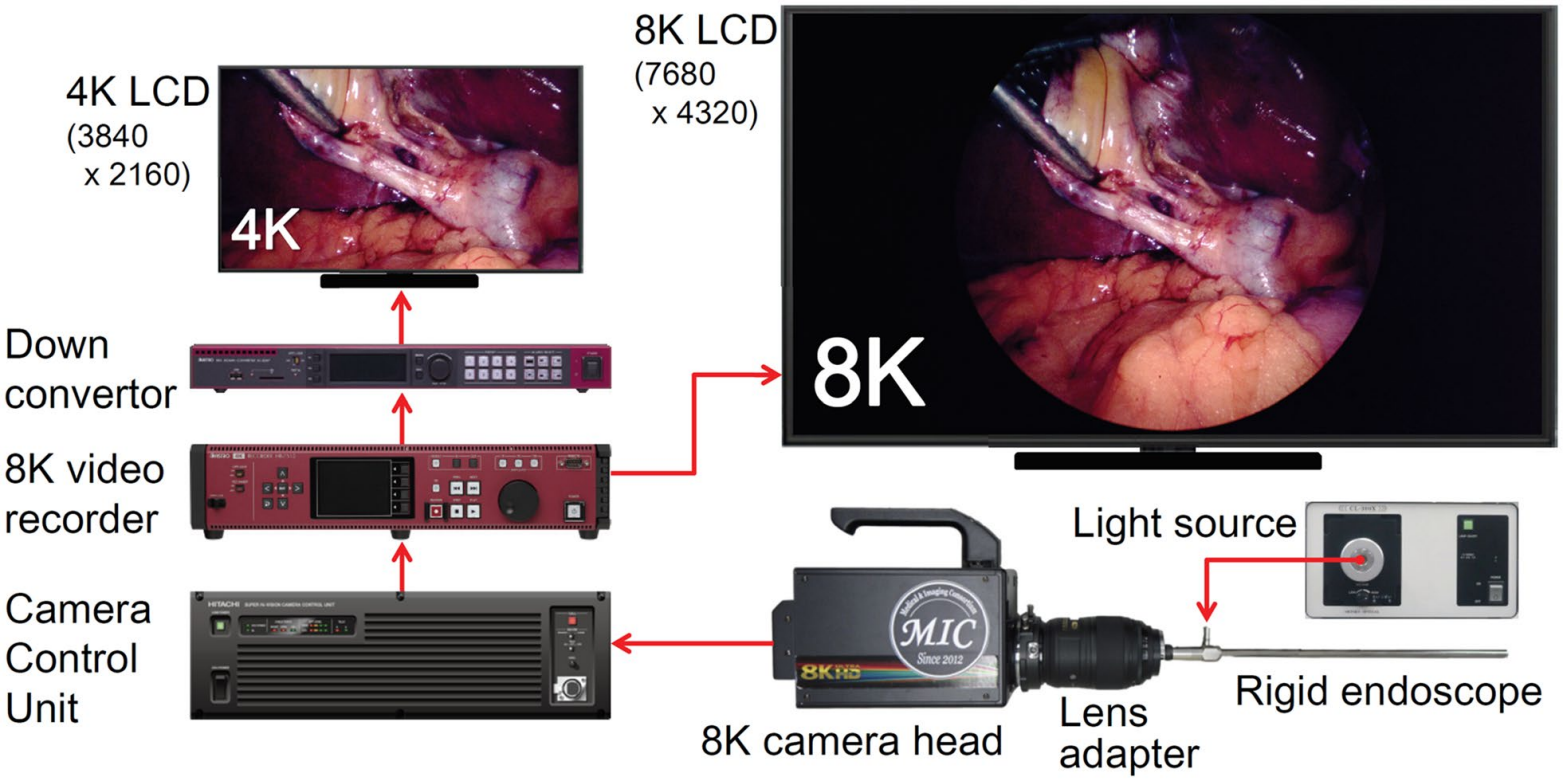
System configuration of the 8K UHD endoscope

Our 8K UHD endoscopic system comprised a 30-degree angled rigid endoscope (SHINKO OPTICAL), an 8K camera head (NHK Engineering System, Inc. and Hitachi Kokusai Electric Inc.), an optimal lens adapter to adjust the range of endoscopic view, a xenon light source (CL-300X, SHINKO OPTICAL), a camera control unit (CCU) (NHK Engineering System, Inc. and Hitachi Kokusai Electric Inc.), an 8K video recorder (HR-7512-A, ASTRODESIGN Inc.,), an 8K liquid crystal display (LCD) (ASTRODESIGN Inc.,) and an extra 4K LCD (DM-3412, ASTRODESIGN Inc.,) with a down converter (SC-8207, ASTRODESIGN Inc.) for monitoring in an another room (Fig. 1).

**Fig. 2:**
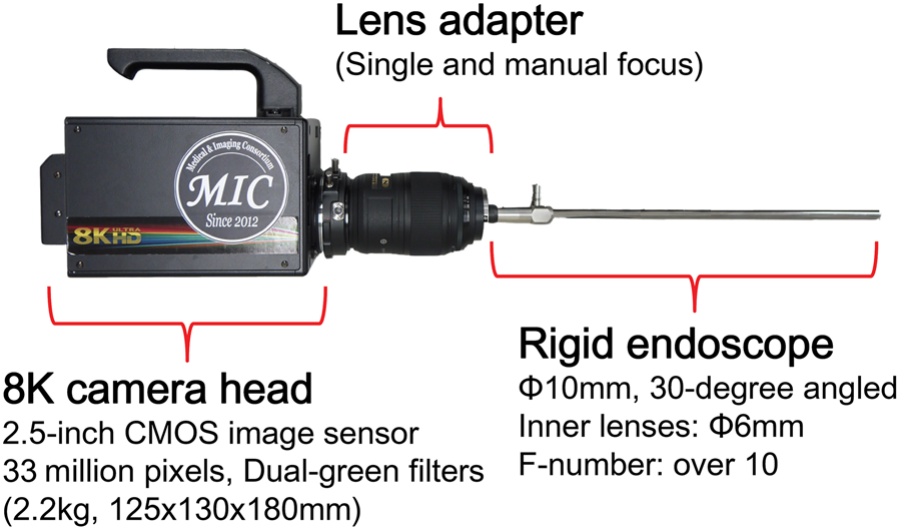
Core part of the 8K UHD endoscopic system, which comprises an 8K camera head, a lens adapter and a rigid endoscope

The core part of the system is shown in Fig. 2. The outer diameter of the rigid endoscope was 10 mm and inner relay lenses of 6 mm in outer diameter were incorporated in the endoscope. These relay lenses were specially selected for 8K imaging and f-number of the rigid endoscope was over 10. The 8K camera head had a single-chip 2.5-inch complementary metal oxide semiconductor (CMOS) image sensor with an 8K resolution and with dual-green color filters (Red × 1, Green × 2, Blue × 1) (Funatsu 2013). The dimensions of the 8K camera head were 125 × 130 × 180 mm and its weight was 2.2 kg. A handle was attached on the outer case of the camera head for ease of holding with a single hand. A lens adapter was mounted between the rigid endoscope and the 8K camera head to enlarge the endoscopic field of view. A standard type of a 300 W xenon lamp for conventional endoscopes was used as the light source. The CCU was equipped with a remote controller to calibrate the color balance and gain in real time. The 8K video recorder had 8 TB of solid state drive (SSD) storage for recording a maximum of 50 min of 8K video. The screen size of the 8K LCD was 85 inches with a resolution of 7680 × 4320 pixels and its color depth was 10 bits.

## 8K imaging resolution performance test

We tested the imaging resolution of our 8K camera head and the rigid endoscope in order to select the optimal lens adapter for 8K imaging using an ISO resolution chart for electronic still cameras. This resolution chart was prepared for the work of ISO TC42 WG18 and is based on ISO 12233. We captured images of the chart and visually counted the black and white lines on the display. At first, we observed the resolution of the display showing the output by the 8K camera itself with an AI Nikkor 50 mm f/1.2S lens (NIKON CORP.). Next we observed the resolutions of reproduction on the display when showing the output as an 8K UHD endoscope with the rigid endoscope with the lenses AF-S Micro NIKKOR 60 mm f/2.8G ED, AI Nikkor 50 mm f/1.2S or AI AF Nikkor 24 mm f/2.8D (NIKON CORP.).

### Clinical cases of cholecystectomy

**Fig. 3:**
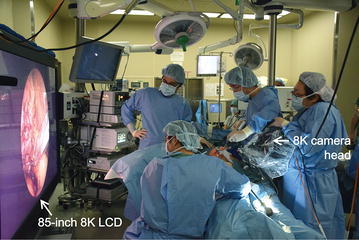
A side view of 8K UHD endoscopic surgical setting

**Fig. 4:**
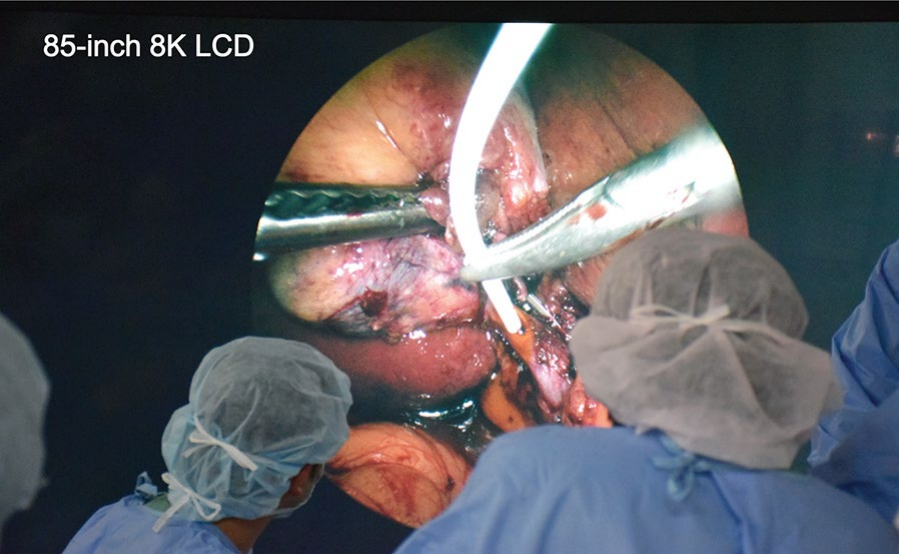
A front view of 8K LCD which is right in front of surgeons

**Fig. 5:**
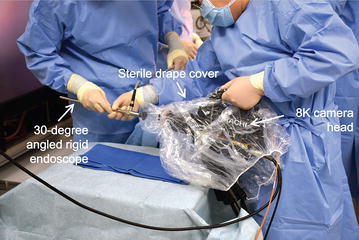
The 8K camera head covered with a sterilized drape before attachment of the rigid endoscope

We employed the 8K UHD endoscopic system in two cases of cholecystectomy in Kyorin University Hospital. The 8K LCD was placed adjacent to the operating table so that the surgeon could stand near, and in front of, the 8K LCD as much as possible because the 8K image is viewed with the best quality when it is viewed in front from a distance of 0.75 times the height of the display (Figs. 3, 4). In this study, we also set up a standard 2K rigid endoscopic system (Karl Storz Endoscope) as a backup to the 8K UHD endoscopic system. Before attachment to the rigid endoscope, the 8K camera head was covered by a sterilized drape since the camera head was not sterilizable (Fig. 5).

The first patient was a male in his seventies and visited Kyorin University Hospital with a complaint of biliary colic. The second patient was also a male in his seventies and visited Kyorin University Hospital with a complaint of abdominal pain and diagnosed with choledocholithiasis.

## Results

### 8K imaging resolution performance

The imaging resolution of the display showing the output by the 8K camera itself with AI Nikkor 50 mm f/1.2S (NIKON CORP.) was about 4000 line widths per picture height on the 8K display, which indicated an intrinsic performance of 8K. The resolutions of reproductions on the display when showing the output as an 8K UHD endoscope with the rigid endoscope were 2200 line widths per picture height with AF-S Micro NIKKOR 60 mm f/2.8G ED, 2600 lines with AI Nikkor 50 mm f/1.2S and 3600 lines with AI AF Nikkor 24 mm f/2.8D. With AI AF Nikkor 24 mm f/2.8D we could attain higher resolution but with a smaller field of view. For laparoscopic surgery, the field of view is required to be as large as possible. Therefore, with priority given to a greater field of view, in this study we selected AF-S Micro NIKKOR 60 mm f/2.8G ED as the lens adapter.

### Clinical findings

In surgery of the first case, since the brightness of the laparoscopic view was insufficient with only a 300-W xenon light source, we utilized the backup 2K rigid endoscope with a 300-W xenon light source as a second light source for illumination. In addition to achieving nearly the standard brightness of the laparoscopic image, we also increased the gain by 9 dB.

We succeeded in completing the two cases of cholecystectomy within the standard surgical time without switching to the standard 2K rigid endoscopic system. From the first and second patients, several gallstones with maximum dimensions of 18 mm and of 5 mm, respectively, were removed. Both patients left the hospital on the fourth postoperative day with the standard prognosis of Kyorin University Hospital.

The surgeons and other surgical staff reported the following positive evaluations of our 8K UHD endoscope.

- With sufficient illumination, laparoscopic images with quite high resolution could be viewed
- 8K laparoscopic images were excellent in reproducing the appearances of solidity and reality
- Vessels on the multilayer membrane around the bile duct and the gallbladder could be clearly recognized
- Viewing of 8K laparoscopic images caused less eye strain.

On the other hand, the following negative evaluations were also reported.

- Without sufficient illumination, the darkness and low quality of color reproduction of the images made laparoscopic surgery impossible
- The large size of the 8K camera head interfered with the surgical field
- The weight of the heavy 8K camera head made it difficult to hold in position even by two laparoscopists.

**Fig. 6:**
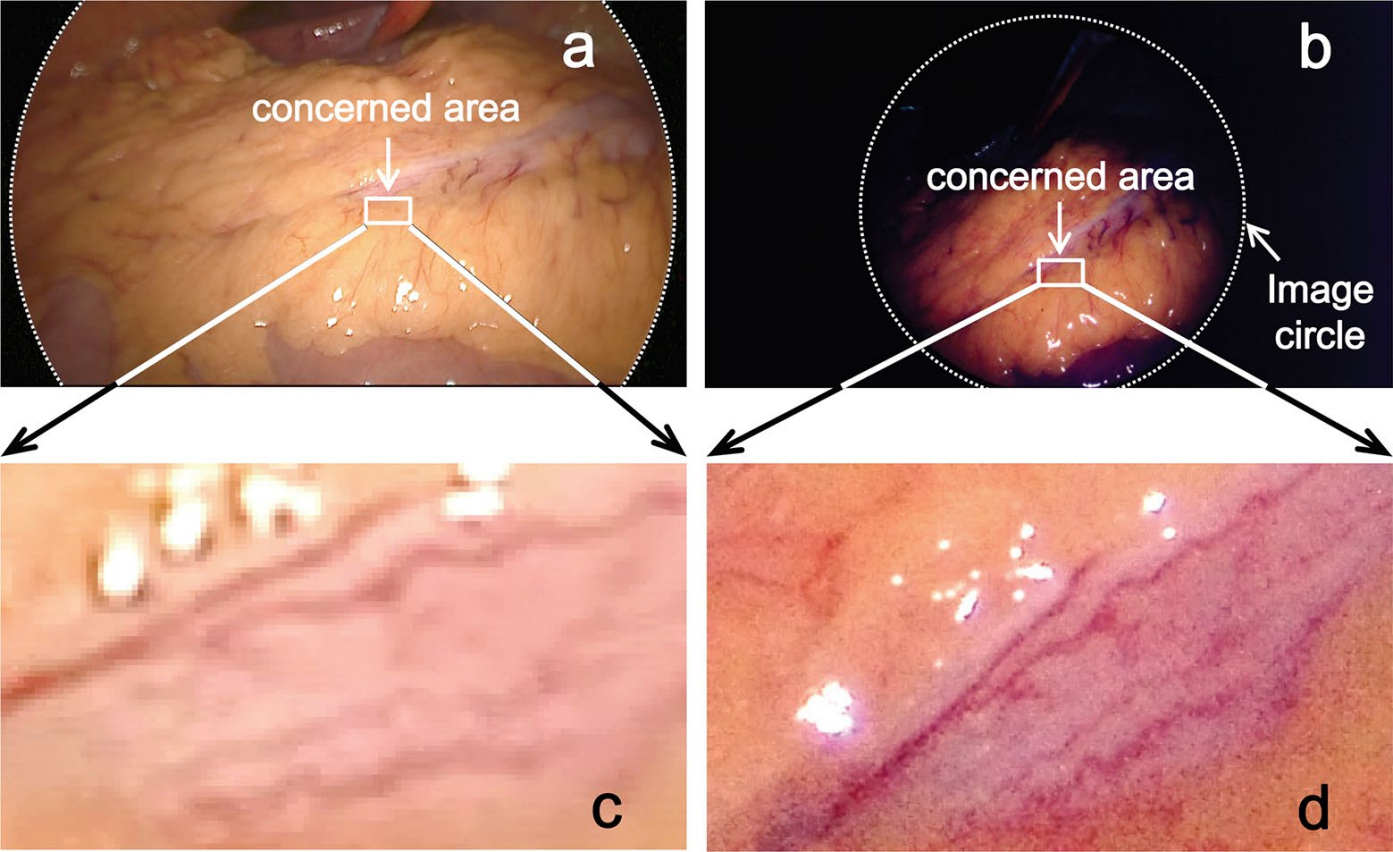
Comparison between 8K and 2K endoscopic images of capillaries on the surface of the large intestine (The 8K UHD endoscopic image is only a print on paper and gives a poor representation of the real 8K resolution. The benefits of 8K resolution can only be seen using the special screen). **a** 2K endoscopic image. **b** 8K endoscopic image. **c** 256-times magnified image of a part of the 2K endoscopic image. **d** 256-times magnified image of the same part of the 8K endoscopic image

We show examples of images of capillaries on the surface of the large intestine for comparison between our 8K endoscope and a conventional 2K endoscope (Fig. 6). In the 256-times magnified 2K image individual pixels are visible and the outlines of the vessels are vague. In the 256-times magnified 8K image, the vessels are somewhat dark but clearly seen.

## Discussion

We achieved clinical success in the performance of 8K endoscopic cholecystectomy. In the experimental and clinical studies using our custom-built 8K endoscopic system, we were able to demonstrate the technological feasibility and clinical utility of the 8K UHD endoscope. 8K endoscopic imaging, compared to imaging by 2K imaging technology, is likely to lead to major changes in future medical practice in terms of the following viewpoints. First, 8K imaging displays true-to-life images of high reality that is able to reproduce the sense of looking onto the original field of view. Second, the 8K UHD endoscope enables reproduction with high resolution corresponding to super visual acuity (around 4.3) with pin-sharp, non-blurred images that make it possible to distinguish diverse tissue architectures which otherwise appear similar to each other and are easily confused. Notably, the spatial resolution was substantially higher in both the central and pericentral areas. This was noted in all areas of the display (85-inch) even near the periphery. Third, a particularly interesting point is that the 8K UHD endoscope with a built-in digital zoom function can be positioned to stay high up close to the abdominal wall of the intraperitoneal cavity, presenting a bird’s eye view of the operative field. This means that the tip of the endoscope does not need to come close to the organs and/or tissues undergoing surgery. Such positioning of the tip of the endoscope is expected to be effective in preventing the surgical devices and the endoscope from clashing into each other, allowing the surgeon more room to easily perform surgical maneuvers.

In conclusion, the 8K UHD endoscopy promises to open up new possibilities for intricate procedures including anastomoses of thin nerves and blood vessels as well as more confident surgical resections of a diversity of cancer tissues. As a result of the experimental and clinical studies using our custom-built 8K UHD endoscope, we have demonstrated the mechanical and engineering feasibility of the 8K UHD endoscope and its clinical utility. 8K endoscopic imaging, compared to imaging by the current 2K imaging technology, is very likely to lead to major changes in the future of medical practice.
